# MicroRNA-378 enhances inhibitory effect of curcumin on glioblastoma

**DOI:** 10.18632/oncotarget.17881

**Published:** 2017-05-16

**Authors:** Wende Li, Weining Yang, Yujiao Liu, Siyu Chen, Shanmin Chin, Xiaolong Qi, Yingchao Zhao, Hao Liu, Jiasheng Wang, Xueting Mei, Peigen Huang, Donghui Xu

**Affiliations:** ^1^ Laboratory of Traditional Chinese Medicine and Marine Drugs, School of Life Sciences, Sun Yat-Sen University, Guangzhou 510275, China; ^2^ Edwin L. Steele Laboratory, Massachusetts General Hospital, Harvard Medical School, Boston, MA 02114, USA; ^3^ Sunnybrook Health Sciences Centre and Department of Laboratory Medicine and Pathobiology, University of Toronto, Toronto, ON, Canada; ^4^ Guangdong Laboratory Animals Monitoring Institute, Guangdong Key Laboratory of Laboratory Animals, Guangzhou 510663, China; ^5^ Cancer center, Union Hospital, Tongji Medical College, Huazhong University of Science and Technology, Wuhan 430022, China

**Keywords:** miR-378, glioblastoma, U87, curcumin

## Abstract

Glioblastoma multiforme is the most aggressive and common primary brain tumor, and is virtually incurable due to its therapeutic resistance to radiation and chemotherapy. Curcumin is a well-known phytochemical exhibiting antitumor activity on many human cancers including glioblastoma multiforme. Given the unique miRNA expression profiles in cancer cells compared to non-cancerous cells, we investigated whether these miRNA could be used to cancer therapy. In this report we show that miR-378, a glioblastoma multiforme down regulated miRNA, may enhance the inhibitory effect of curcumin on this cancer growth. Our results indicated that the inhibitory effect of curcumin was enhanced in miR-378-expressing stable U87 cells *in vitro* and *in vivo,* compared to control cells. MiR-378 was found to target p-p38 expression, underlying the observed phenotypic changes. Thus, we concluded that miR-378 enhances the response of glioblastoma multiforme to curcumin treatment, by targeting p38.

## INTRODUCTION

Glioblastoma multiforme (GBM) is the most common and most aggressive malignant brain tumor that accounts for 40% of all brain tumors, with only a about 3% five-year survival rate [1]. It is difficult to treat surgically, or with chemotherapy or radiotherapy, given its highly proliferative and invasive nature [2]. Little progress has been achieved in the treatment of GBM over the last twenty years, partly due to a poor mechanistic understanding of GBM, and thus a lack of effective therapeutic targets [3].

MicroRNAs (miRNAs) are small non-coding RNA molecules containing about 22 nucleotides that regulate gene expression at the post-transcriptional level. Some of the miRNAs are expressed specifically in the mammalian brain and retina, indicating that this group of miRNAs might be involved in the development of neural tissues [4, 5]. Moreover, since unique microRNA expression profiles have been frequently identified in various cancer cells, such as lung cancer [6] and cervical cancer [7]. Abnormal expression of miR-378 has been reported in many cancer cell lines, such as K562, Jurkat, and HL-60 [8]. It was also found that miR-378 expression was suppressed in GBM [9]. It has been found miRNAs may influence drug resistance in cancer treatment [10, 11]; hence more and more studies have been launched to investigate the synergistic effects of miRNAs and approved drugs in cancer treatment.

Curcumin is a natural polyphenol compound that has been widely used in the inflammation, respiratory diseases, and cancer therapeutics [12, 13]. It had been reported that curcumin possesses anticancer effects by regulating functions of numerous targets such as nuclear factor NF-κB, extracellular signal-regulated kinase (ERK), and cell cycle proteins [14, 15]. It has also been demonstrated that curcumin might inhibit multiple matrix-metalloproteinases, suppressing GBM proliferation, and inducing apoptosis or autophagy [16, 17].

In this study we examined whether miR-378 may influence the effect of curcumin on GBM and we found that miR-378 enhanced the response of Glioblastoma cells to curcumin treatment.

## RESULTS

### miR-378 potentiates inhibitory effect of curcumin on GBM growth

**Figure 1:**
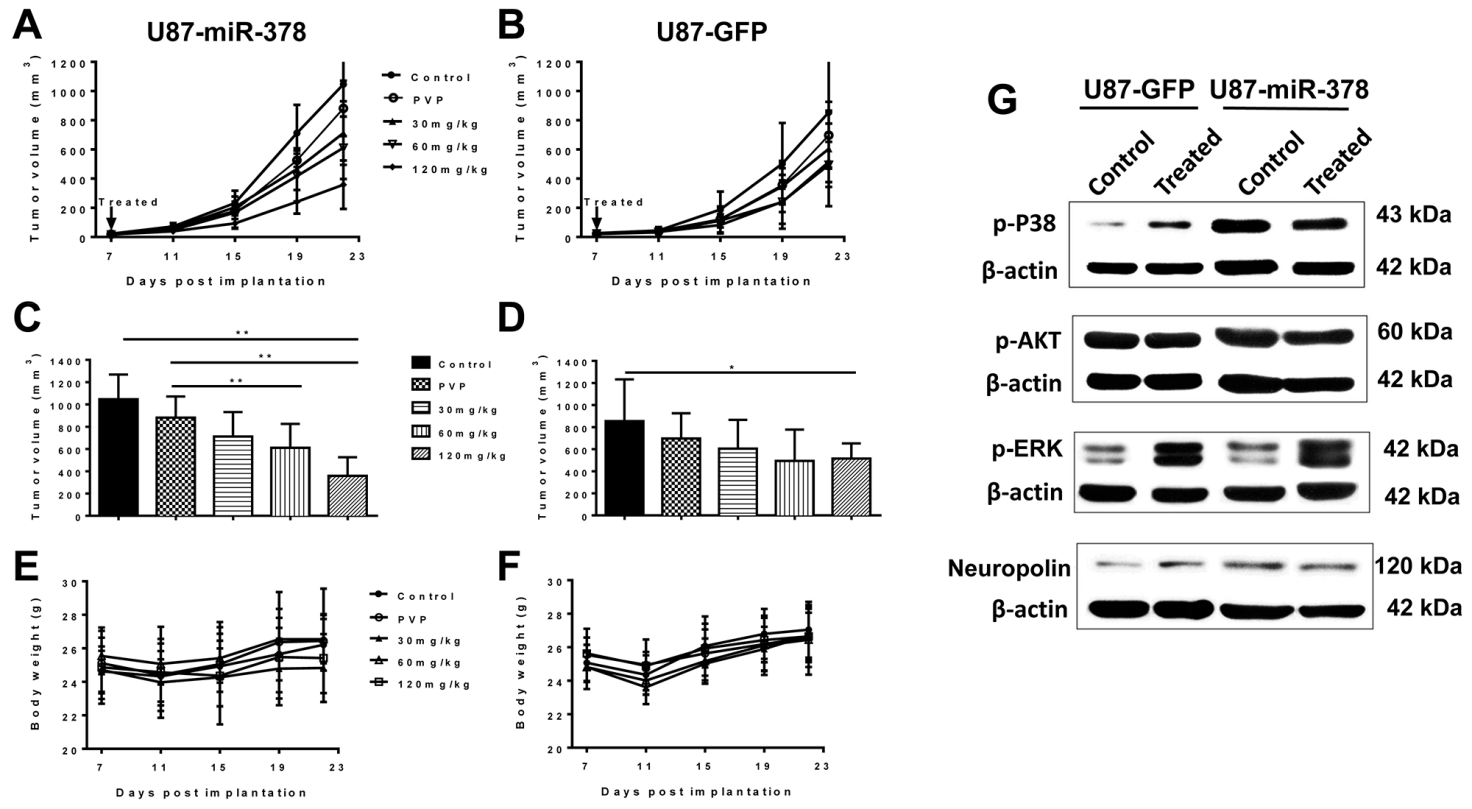
MiR-378 potentiates inhibitory effect of curcumin on GBM growth (**A-F**): Curcumin-PVP (Polyvinylpyrrolidione) was diluted into 120 mg/kg, 60 mg/kg and 30 mg/kg, and PVP concentration equal to that in 120 mg/kg curcumin-PVP. Source tumors were initiated from *in vitro* cultured cells (U87-miR-378 and U87-GFP cells). Experimental tumors were initiated by subcutaneous chunk transfer of source tumors to the hind leg of 6-7 weeks old male SCID mice provided by the Cox-7 defined flora animal facility in MGH. After 7 days post implantation, all of the SCID mice were randomly grouped into Control (only PBS), PVP, 30 mg/kg, 60 mg/kg and 120 mg/kg groups. Each group accepted daily gavage (1 time/mouse/day) for 2 weeks, and the tumors were harvested after last 24h treatment. Data are mean± SD (n=3), **p<0.01, the results were repeated in three independent experiments. (**G**): Western blot analysis of p-P38, p-AKT, p-ERK and Neuropolin expressed in U87-miR-378 and U87-GFP tumors. β-actin was used as loading control.

The effects of curcumin on SCID mice xenograft tumor model were first examined. Both U87-miR-378 and control U87-GFP cells formed obvious tumors at Day 11 post-injection (Figure 1A & 1B). Mouse body weight was not affected by drug treatment (Figure 1E & 1F). According to the results, the growth of U87-miR-378 tumors (Figure 1A & 1C) was significantly inhibited by 60 mg/kg curcumin (611.2 ± 214.6, P<0.01) and 120 mg/kg curcumin (358.8 ± 166.5, P<0.01) when compared with the PVP (Polyvinylpyrrolidione) group (881.32 ± 189.84) after 2 weeks of treatment. There was no significant difference between the PVP group and PBS groups (1046.5± 221.9, P>0.05). There was also no statistically significant difference in the 60 mg/kg group (494.4 ± 283.1, P>0.05) or 120 mg/kg group (514.5 ± 138.3, P>0.05) when compared with PVP group (697.4 ± 228.8) (Figure 1B & 1D). Our *in vivo* study indicated that 60 mg/kg and 120 mg/kg curcumin was sufficient to suppress tumor growth induced by miR-378 expression.

To investigate the mechanism underlying the interaction between miR-378 and curcumin, western blot was performed to determine the expression of phosphorylation extracellular signal-regulated kinase (ERK), Akt and P38, as well as neuropilin in mice (Figure 1G). We found that p-p38 was up-regulated in U87-miR-378 cells formed tumor compared to that of U87-GFP cells.

### miR-378 enhances the effect of curcumin by suppressing cell proliferation and inducing apoptosis.

**Figure 2:**
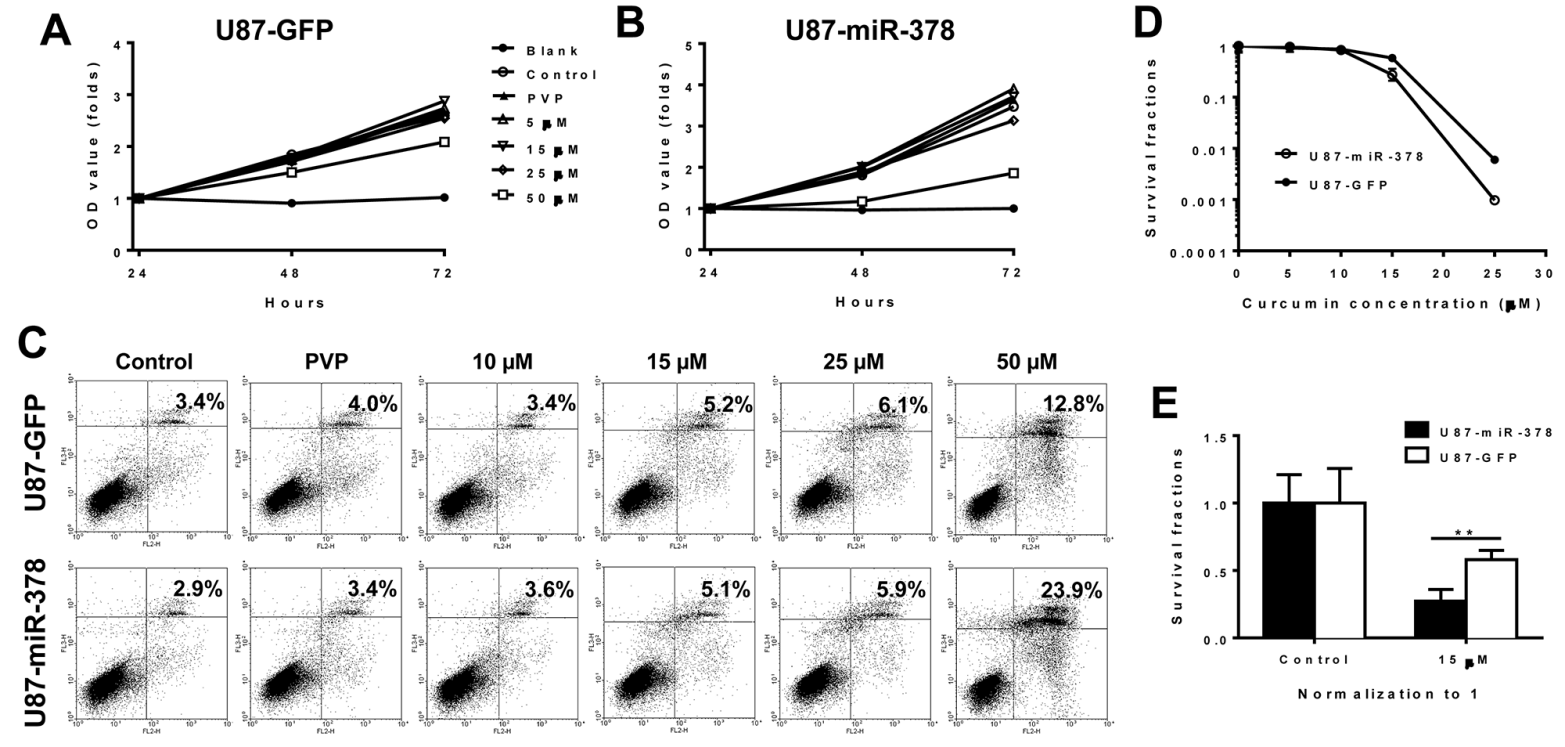
MiR-378 enhances the effect of curcumin by suppressing cell proliferation and inducing apoptosis (**A-B**): Effects of different concentration curcumin on the growth of U87-GFP and U87-miR-378 cells by MTT assay. U87-GFP and U87-miR-378 cells were treated with 0 μM, 5 μM, 15 μM, 25 μM and 50 μM curcumin respectively, the tested cells were seeded at density 2000 cells/well and measured in 24 h, 48 h and 72 h after culture. The experiments were repeated three times and the negative control was conducted using only cell-free culture medium (means ± SD). Data are mean± SD (n=3), **p<0.01, the results were repeated in three independent experiments. C: Effects of different concentration curcumin on the cell apoptosis of U87-GFP and U87-miR-378 cells by Flow assay. (**D-E**): Effects of different concentration curcumin on U87-GFP and U87-miR-378 cells by colony formation assay. The surviving fraction was calculated as a ratio of the number of colonies formed and the product of the number of cells plated and the plating efficiency. The results were repeated in three independent experiments.

U87 cells over expressing miR-378 were used to investigate whether miR-378 could influence the inhibitory effect of curcumin on cell proliferation. Stable U87-GFP and U87-miR-378 cells were treated with 0 μM, 5 μM, 15 μM, 25 μM and 50 μM curcumin-PVP. The effects of curcumin treatment on cell proliferation in U87-GFP cells were consistent with our previous report (Figure 2A). Interestingly, we found that 50 μM curcumin demonstrated higher inhibition on U87-miR-378 proliferation than on U87-GFP (P<0.05) after 48 h and 72 h treatments (Figure 2B).

Moreover, 72-hour treatment with 50 μM curcumin caused more cell apoptosis in U87-miR-378 compared with that in 23.9% vs. 12.8% (Figure 2C). The results suggested that the inhibitory effect of miR-378/curcumin observed in MTT assay (Figure 2A & 2B) was due to apoptosis but not other factors, such as change of cell cycle duration.

To examine whether U87-miR-378 and U87-GFP cells behaved differently in forming colonies under curcumin treatment, the cells were grown in medium supplemented with 0 µM, 5 µM, 10 µM, 15 µM, 25 µM curcumin, and colony formation assay was performed. This was designed to test whether U87-miR-378 cell were less aggressive than U87-GFP cells. Results indicated that more colonies were formed by U87-GFP cells compared to U87-miR-378 cells, following 15 µM or 25 µM curcumin treatment (Figure 2D). Furthermore, the survival rate was not significantly different between the two cells at 5 µM and 10 µM curcumin treatment, but was significantly different at 15 µM (P<0.01, U87-miR-378 VS U87-GFP) (Figure 2E) and 25 µM (P<0.01, U87-miR-378 VS U87-GFP, data not show here). All of the results indicated that the U87-miR-378 cell line was more sensitive to 15 µM and 25 µM curcumin treatment than U87-GFP.

**Figure 3:**
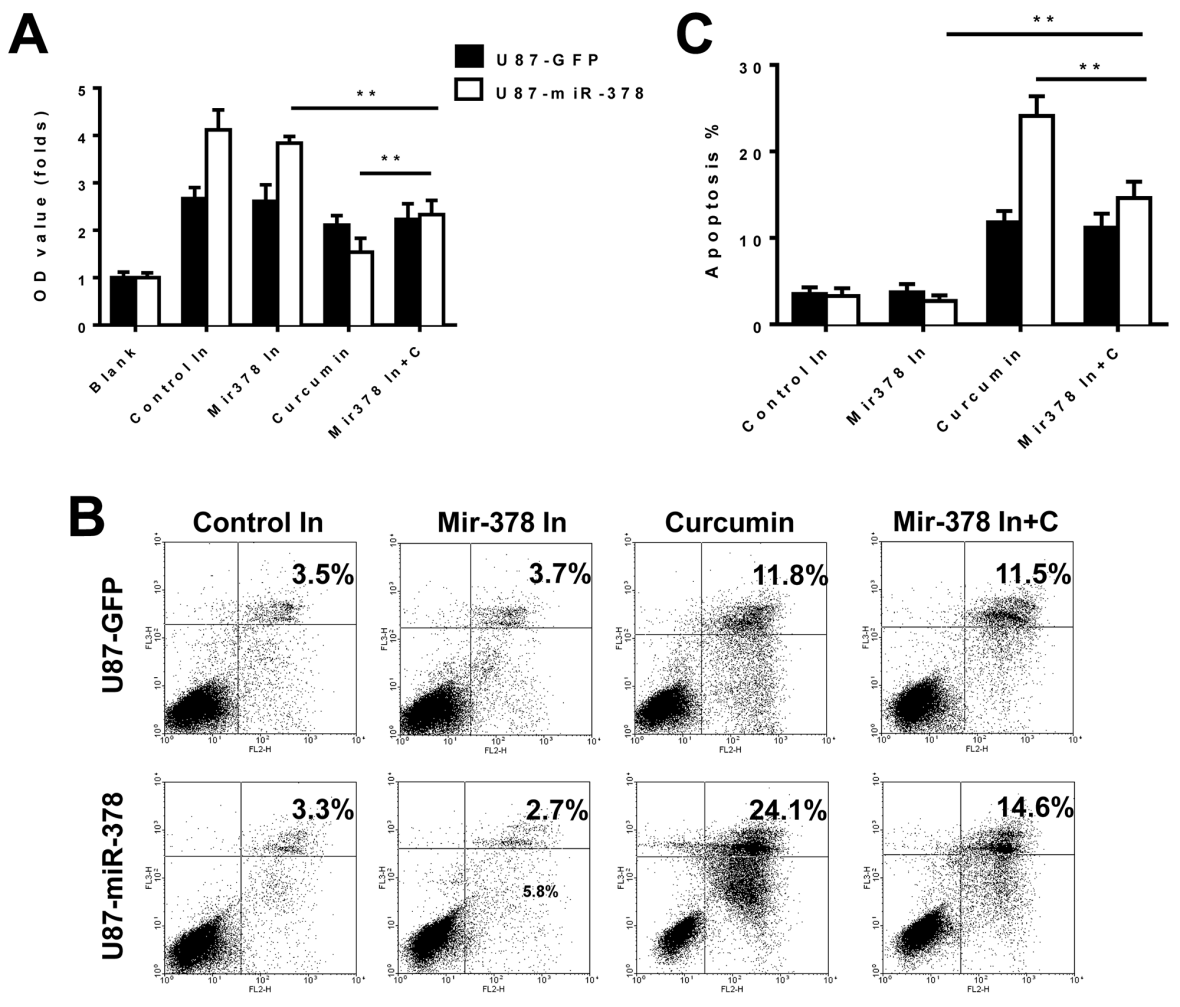
MiR-378 enhanced the sensitivity of curcumin treatment in U87 cells Effects of miR-378 inhibitor, curcumin and miR-378 inhibitor on the growth of U87-GFP and U87-miR-378 cells by MTT assay (**A**) and flow cytometer (**B, C**). There is significantly difference between U87-GFP and U87-miR-378 cells in the curcumin group and miR-378 inhibitor with curcumin group. The t-test was used for statistical analysis. Data are mean± SD (n=3), **p<0.01, the results were repeated in three independent experiments. Control In: negative miRNA-378 inhibitor; miRNA-378 In: miRNA-378 inhibitor; C: curcumin.

To confirm our results, we used a loss-of-function approach. A miR-378 inhibitor was transfected into the cells to confirm the functions of miR-378 described above. MTT and apoptosis assays showed that miR-378 enhanced the sensitivity of curcumin treatment in U87 cells (Figure 3).

### miR-378 enhances U87 cell line responses to curcumin treatment via stimulating P38 signaling pathway

**Figure 4:**
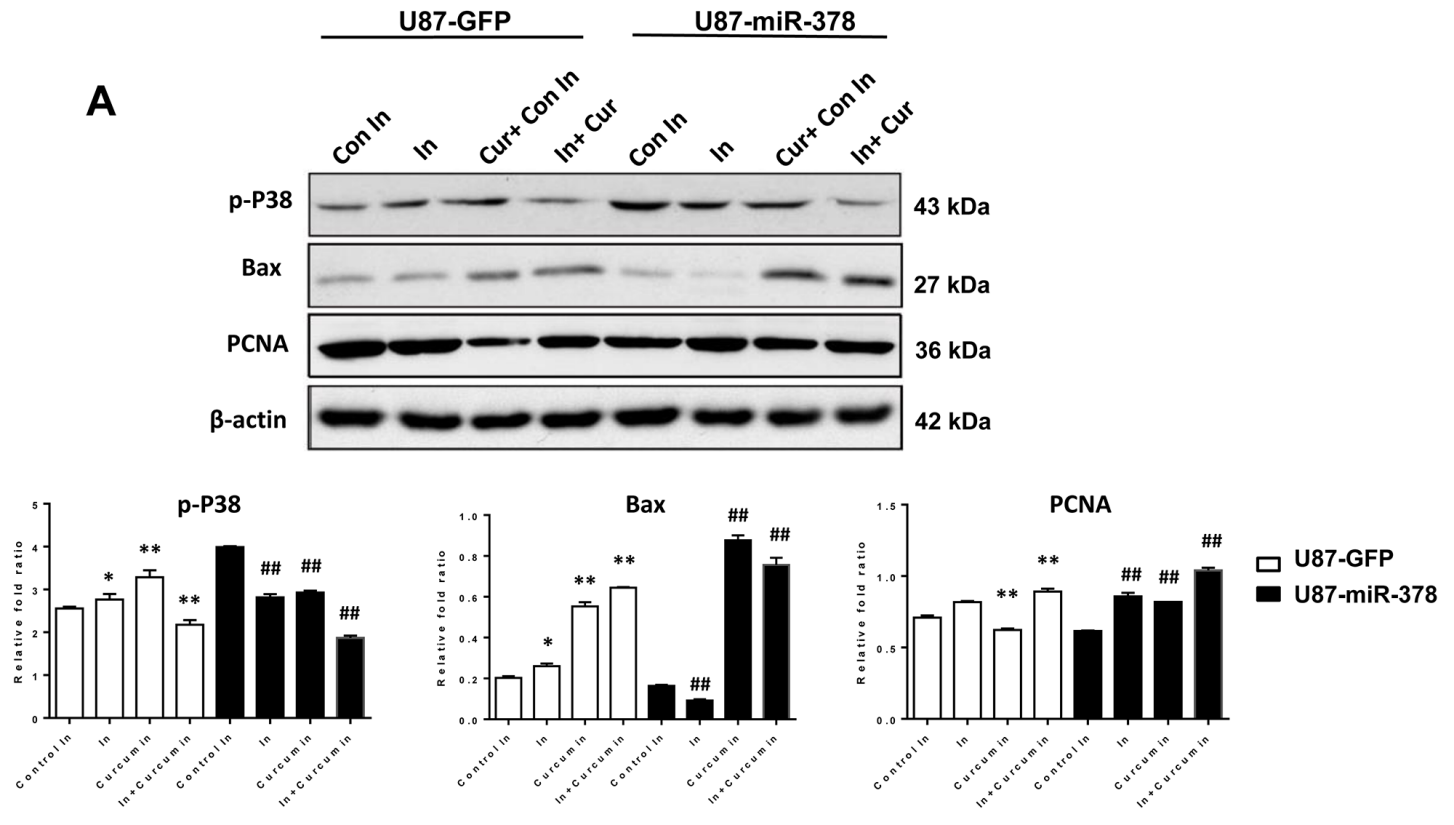

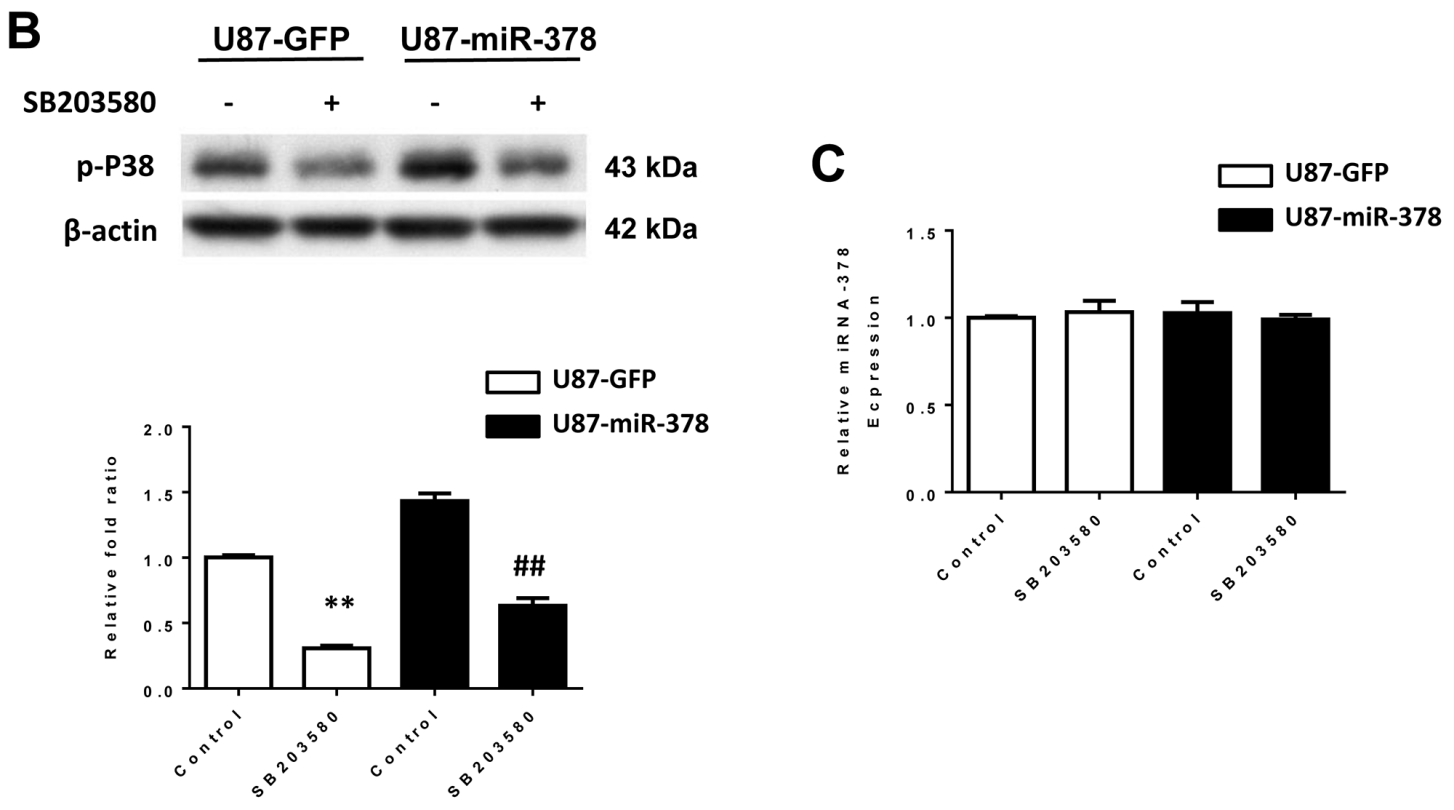
MiR-378 enhanced the sensitivity of curcumin treatment in U87 cells by p38 signaling pathway (**A**): Western blot analysis of p-P38, Bax, PCNA expressed in U87-miR-378 and U87-GFP cells with or without curcumin and miR-378 inhibitor treated. Con In: control miRNA-378 inhibitor; In: miRNA-378 inhibitor; Cur:curcumin. β-actin was used as loading control. The t-test was used for statistical analysis. Data are mean± SD (n=3), the results were repeated in three independent experiments. *p<0.05, **p<0.01 (vs U87-GFP control miRNA-378 inhibitor); ^#^p<0.05, ^##^p<0.01 (vs U87-miRNA-378 control miRNA-378 inhibitor). (**B**): Western blot analysis of p-P38 expressed in U87-miR-378 and U87-GFP cells with or without SB203580 (P38 inhibitor) treated. β-actin was used as loading control. The t-test was used for statistical analysis. Data are mean± SD (n=3), the results were repeated in three independent experiments. **p<0.01 (vs U87-GFP control), ^##^p<0.01 (vs U87-miR-378 control). (**C**): qRT-PCR analysis of miRNA-378 levels in U87-miR-378 and U87-GFP cells with or without SB203580 (P38 inhibitor) treated. The t-test was used for statistical analysis. Data are mean± SD (n=3), the results were repeated in three independent experiments.

Since miR-378 was reported to be able to regulate Mitogen-Activated Protein Kinase Pathway (MAPK) [18], we next examined whether the MAPK pathway had been affected by the miR-378/curcumin axis. It was been found that curcumin suppressed the protein levels of phosphorylated p38 in U87 cells (Figure 4A). miR-378 could counteract the inhibition of p38 by increasing phosphorylation of p38, thereby increasing the sensitivity of cells to curcumin. There was not significant affection in miR-378 level after treated by p38 inhibitor (SB203580) (Figure 4B & 4C). The phosphorylation of p38 led to high expression of Bax, indicating the increase of apoptosis. The insertion of Bax into the mitochondrial membrane induces the release of cytochrome C and the induction of apoptotic cell death. Furthermore, low expression of PCNA was detected after curcumin treatment, indicated reduced cell proliferation in U87 cells. PCNA expression may be used as a marker of cell proliferation. Collectively, these data suggested that miR-378 enhanced U87 cell line responses to curcumin treatment via stimulating the P38 signaling pathway.

## DISCUSSION

GBM is one of the most common tumors with a relatively low survival rate. Due to its highly proliferative and invasive nature, finding agents to increase the therapeutic efficiency of established drugs is a feasible therapeutic strategy. miRNAs, as a novel biomarker, have been widely used to predict prognosis and treatment efficiency. Nearly 1900 human miRNAs have been identified, which can regulate 60∼80% of the genes in humans [19]. miR-34a, miR-137, and miR124 have been reported to play roles in the maintenance of cancer stem cell properties [20, 21]. miR-21 and miR-196 has been shown to be over-expressed in glioblastoma cell lines and glioblastoma patients [22, 23]. miR-138 inhibits GBM cell proliferation *in vitro* and tumorigenicity *in vivo* through inducing cell cycles G1/S arrest [24]. These studies shed light on the possibility of targeting miRNA as a direction for gene therapy.

Curcumin is a polyphenolic agent with anti-inflammatory and anticarcinogenic properties, which is known to induce apoptotic activity against a variety of cancers, such as stomach cancer, colon cancer, breast cancer and prostate cancer [25–27]. It can also inhibit multiple matrix-metalloproteinase and suppress the growth of GBM cell in a dose-and time-dependent manner [28]. Our study mainly focuses on whether miR-378 could enhance U87 cell line response to curcumin therapy. We found that miR-378 significantly enhanced the effect of 50 µM curcumin treatment by suppressing the proliferation of U87 cells (Figure 2). In addition, colony formation assay was performed following curcumin treatment in U87-GFP and U87-miR-378 cells (Figure 2). The result showed that the survival fraction was significantly different in 15 µM dose and 25 µM doses (U87-GFP vs U87-miR-378 cell), which indicated that the U87-miR-378 cell was more sensitive to curcumin treatment. Consistent results were also obtained in *in vivo* xenograft tumor model (Figure 1). 60 mg/kg and 120 mg/kg curcumin could suppress tumor growth formed by U87-miR-378 cells to a greater extent compared with control U87-GFP cells.

The mechanism of miR-378 enhanced curcumins effects in U87 cells was also investigated by western blot (Figures 1 and 4). Our data revealed that miR-378 acquired tumor inhibition with curcumin through targeting p-p38. Thus, we infer that the reduction in the growth of U87 may be caused by decreases phosphorylation of p38, which was curbed by miR-378.

p38 activity contributes to the highly invasive capacity of GBM cell lines and regulates the expression of MMPs, inflammation, and other cell process, making it a suitable drug target [29, 30]. A number of studies have demonstrated that the p38 MAPK signaling pathway is involved in apoptosis in many cell types [31–33]. According to the results shown in Figure 4, the phosphorylation of p38 leads to increased Bax expression, and GBM cell apoptosis. These results are consistent with previous reports [34]. Furthermore, we found the decrease of PCNA after curcumin treatment, which might suggest the inhibition of cell proliferation in U87.

MiR-378 has emerged as molecular regulators that play key roles in pathogenesis and progression of different maliganceise. miR-378a-3p could suppresses activation of hepatic stellate cells by targeting Gli3 and its expression is regulated by Smo-dependent NF-ΚB signaling, suggesting miR-378a-3p has therapeutic potential for liver fibrosis [35]. MiR-378 could directly target secreted clusterin and help disable the chemoresistance against cisplantin in lung adenocarcinoma cells [36]. Besides, miR-378 also involved in breast cancer, Browne G et al identifies a novel and clinically relevant mechanism for regulation of Runx1 in breast cancer that is mediated by PPARGC1B-miR-378-Runx1 regulatory pathway [37]. overexpression of miR-378 suppressed prostate cancer cell migration and invasion promoted cell apoptosis and stably miR-378-overexpressed prostate cancer cells displayed a significantly reduction in tumor growth [38]. Over-expression of miR-378 inhibits glioma cell migration and invasion [9]. In addition, the expression level miR-378 was significantly lower in glioma tissues compared with non-neoplastic brain tissues. Patients with lower miR-378 expression levels also have a significantly poorer overall survival [39]. Li et al demonstrated that miR-378 functioned as a tumor suppressor and played an important role in inhibiting tumor migration and invasion [9]. All those findings are consistent with the results of our work and strongly support that fact that miR-378 may synergistically act with curcumin in inhibiting the growth of glioblastoma cells.

Thus, we conclude that miR-378 acts as a potential tumor suppressor in GBM, a function that is accomplished by target p38. miR-378 could enhance the sensitivity of curcumin treatment in U87 cells, which can help us solve the toxicity of high-dose curcumin. Collectively, our results together with previous reports clearly suggest that miR-378 plus curcumin could be a useful combination for the treatment of GBM. In the future, studies on the regulation of miR-378 promoter may help to identify small molecule drugs that may induce the endogenous expression of miR-378. An improved understanding of the interplay between microRNAs and therapeutic agents will allow for improved treatment of GBM.

## MATERIALS AND METHODS

### Cell lines and culturing conditions

Human glioblastoma U87 cell lines stably expressing miR-378 (U87-miR-378) or green fluorescent protein as control (U87-GFP) were obtained from Dr. Burton Yang [40]. The cell lines were cultured in DMEM supplemented with 10% fetal bovine serum (Hyclone, Thermo Scientific) at 37°C under humidified atmosphere of 5% CO_2_, 95% air.

### Preparation of curcumin-PVP solid dispersion

Curcumin was purchased from Tianjin Guangfu Fine Chemical Research Institute (Tianjin, China). Ranitidine hydrochloride was purchased from Alpharma Pharmaceuticals Inc. (Piscataway, NJ, USA); the PVP K30 was purchased from BASF Pharmacy Co. Ltd (Ludwigshafen, Germany). The curcumin–PVP solid dispersion were prepared as descripted previously [41]. In brief, 1:6 mixtures of curcumin-SDS and PVP K30 in were produced by conventional solvent evaporation method. Curcumin/PVP K30 were dissolved in a minimum volume of ethanol and the solvent was removed under vacuum in a rotavapor at 65°C and 80 rpm for 8h. The resulted solid dispersion was pulverized with mortar and pestle, passed through a 250-μm sieve (mesh size 60), kept in vacuum-desiccator at 40°C for 24h, and then stored in a desiccator at room temperature.

### Transfection of U87 with miR-378 inhibitor

A total of 2 × 10^5^ cells per well of U87 cells were seeded in 6-well plates and transfected with miR-378 mimic or inhibitor (Ambion, Thermo Scientific, Waltham, MA, USA) following Fast-Forward Transfection protocol (QIAGEN, Hilden, Germany). 3 days post-transfection, the cells were harvested for total RNA isolation or protein extraction.

### Animal experiments

All animal work was conducted following the guidelines of Public Health Service Policy on Humane Care of Laboratory Animals, and approved by the Institution Animal Care and Use Committee at the MGH. 6-7 weeks old male SCID mice were purchased from the Cox-7 animal facility in MGH and used in the experiments. To generating tumor xenografts, 2 million U87-miR-378 or U87-GFP cells were subcutaneously injected into the hind legs of the mice. 7 days post- implantation, the mice were randomly divided into 5 groups PBS, PVP, and curcumin treatment groups with a dose of 30 mg/kg, 60 mg/kg, and 120 mg/kg, respectively. Each group has 7 or 8 animals and the mice received daily gavage (1 time/mouse/day) for 2 weeks. Mouse body weight and tumor growth were monitored 2 times/week during the 2-week treatment.

### MTT cell viability assay

U87-miR-378 or U87-GFP cells were seeded in 96 well plates at 2000/per well and incubated for 24 hours before drug treatment were conducted. When treatments were completed, 20 μl of 3-(4,5-dimethylthiazol-2-yl)-2,5-diphenyltetrazolium bromide (MTT) reagent (5 mg/ml, Sigma-Aldrich) was added to each well and incubated at 37°C for 3h. Then the medium was discarded and 150 μl DMSO (Sigma-Aldrich) was added to each well and incubated for 10 min at room temperature. Absorbance was measured at a wavelength of 570 nm by SpectraMax 250 microplate reader (Molecular Devices Corp, Concord, ON, Canada).

### Apoptosis assay

U87-miR-378 and U87-GFP control cells were harvested by trypsinization and washed once with PBS. 1 X 10^5^ cells were stained with FITC-conjugated annexin V and propidium iodide purchased from Phoenix Flow Systems (San Diego, CA, USA) following manufacturer's instructions. The stained cells were then analyzed on a FACSCalibur flow cytometer (BDIS, Becton Dickinson, San Jose, CA, USA). Data was analyzed with Cell Quest software (BDIS).

### Colony formation assay

Single cell suspensions were prepared, counted, plated into T25 tissue culture flasks. At each dose point, 4-6 flasks containing a 2-4 folds range of test cells were plated. Cells were routinely plated into medium with G418 (400 µg/ml, Fisher BioReagents, Fair Lawn, NJ, USA). Following treatment, the control cells were cultured for 8 days. Cells exposed to high doses were cultured up to 18 days, stained with crystal violet (Sigma-Aldrich). Colonies containing more than 50 cells were counted. The results were repeated in three independent experiments. The surviving fraction (SF) was calculated as a ratio of the number of colonies formed and the product of the number of cells plated and the plating efficiency.

### Western blot

Proteins of cell lysate were separated on a 10-15% SDS-PAGE gel and then transferred onto the Immun-Blot PVDF membrane (Biorad). After blocking, the membrane was incubated overnight at 4°C with 1: 1000 primary antibodies p-P38 (#9211, Cell Signaling), p-AKT (#4060, Cell Signaling), p-ERK (#4370, Cell Signaling), Bax (#2772, Cell Signaling), PCNA (#2586, Cell Signaling), and anti-β-actin antibodies. Neuropilin (#SC554, Santa Cruze) antibody was diluted in 1:200 in 5% BSA. After washing three times with TBST, the membrane was incubated in corresponding secondary antibody (Bio-Rad, Hercules, CA, USA) at 1: 2000 dilutions for another 1 hour. Excess antibody was washed away three times with TBST and signal was developed with ECL western blotting detection system (Amersham Biosciences).

### MiRNA analysis

For miRNA analysis, cDNA synthesis and qPCR was performed using TaqMan miRNA assays according to the manufacturer's recommendations (Applied Biosystems). Experimental groups were analyzed in replicates of six and normalized to U6.

### Statistical analysis

Data are presented as mean ± SD. Comparisons among different groups were performed by t-test with Primer of Biostatistics. P<0.05 was considered statistically significantly.
